# A Parental Smoking Cessation Intervention in the Pediatric Emergency Setting: A Randomized Trial

**DOI:** 10.3390/ijerph17218151

**Published:** 2020-11-04

**Authors:** E. Melinda Mahabee-Gittens, Robert T. Ammerman, Jane C. Khoury, Meredith E. Tabangin, Lili Ding, Ashley L. Merianos, Lara Stone, Judith S. Gordon

**Affiliations:** 1 Division of Emergency Medicine, Cincinnati Children’s Hospital Medical Center, Cincinnati, OH 45229–3026, USA; lara.stone@cchmc.org; 2 College of Medicine, University of Cincinnati, Cincinnati, OH 45267, USA; robert.ammerman@cchmc.org (R.T.A.); jane.khoury@cchmc.org (J.C.K.); meredith.tabangin@cchmc.org (M.E.T.); lili.ding@cchmc.org (L.D.); 3 Division of Behavioral Medicine and Clinical Psychology, Cincinnati Children’s Hospital Medical Center, Cincinnati, OH 45229–3026, USA; 4 Division of Biostatistics and Epidemiology, Cincinnati Children’s Hospital Medical Center, Cincinnati, OH 45229–3026, USA; 5 School of Human Services, University of Cincinnati, Cincinnati, OH 45221, USA; ashley.merianos@uc.edu; 6 College of Nursing, University of Arizona, Tucson, AZ 85721, USA; judithg@arizona.edu

**Keywords:** tobacco, cessation, emergency department, parents, secondhand smoke

## Abstract

We examined the efficacy of a pediatric emergency visit-based screening, brief intervention, and referral to treatment (SBIRT) condition compared to a control condition (Healthy Habits Control, HHC) to help parental smokers quit smoking. We enrolled 750 parental smokers who presented to the pediatric emergency setting with their child into a two-group randomized controlled clinical trial. SBIRT participants received brief cessation coaching, quitting resources, and up to 12-weeks of nicotine replacement therapy (NRT). HHC participants received healthy lifestyle coaching and resources. The primary outcome was point-prevalence tobacco abstinence at six weeks (T1) and six months (T2). The mean (SD) age of parents was 31.8 (7.7) years, and 86.8% were female, 52.7% were Black, and 64.6% had an income of ≤$15,000. Overall abstinence rates were not statistically significant with 4.2% in both groups at T1 and 12.9% and 8.3% in the SBIRT and HHC groups, respectively, at T2. There were statistically significant differences in SBIRT versus HHC participants on the median (IQR) reduction of daily cigarettes smoked at T1 from baseline (−2 [−5, 0] versus 0 [−4, 0], *p* = 0.0008),at T2 from baseline (−4 [−9, −1] vs. −2 [−5, 0], *p* = 0.0006), and on the mean (SD) number of quit attempts at T2 from baseline (1.25 (6.5) vs. 0.02 (4.71), *p* = 0.02). Self-reported quitting rates were higher in SBIRT parents who received NRT (83.3% vs. 50.9%, *p* = 0.04). The novel use of the pediatric emergency visit to conduct cessation interventions helped parents quit smoking. The near equivalent abstinence rates in both the SBIRT and HHC groups may be due to underlying parental concern about their child’s health. Cessation interventions in this setting may result in adult and pediatric public health benefits.

## 1. Introduction

Annually, there are over 30 million emergency departments (ED) visits by children [1] and Urgent Cares (UC) are becoming a common setting for treating children [2]. Parents who visit these settings for immediate care of their child’s nonacute and acute illnesses [3,4,5] are frequently publicly insured or uninsured [1,6,7,8,9]. Research indicates that more than one in three parents who visit the pediatric ED (PED) or UC are active smokers [9,10,11]. Since many adults access healthcare for their children more often than for themselves [12], PEDs/UCs represent largely untapped settings which can be leveraged to provide smoking cessation interventions to parental smokers. Over 50% of parents who present to the PED/UC are motivated to quit, receptive to receiving cessation interventions, and aware that tobacco smoke exposure (TSE) is harmful to their child’s health [9,13,14]. Further, the PED/UC visit may represent a “teachable moment” during which parental smokers may be more receptive to cessation interventions given concern for their child’s acute illness [15,16]. Thus far, past studies have not demonstrated differences in cessation outcomes as a result of parental smoking cessation interventions that are delivered during ill visits compared to well visits [17].

The adult ED has offered tobacco control interventions for decades [18,19]. Components of ED interventions include provision of printed materials, motivational interviewing (MI)-based cessation counseling, post-visit phone calls, and pharmacotherapy [18,20,21,22]. Overall, these interventions are effective in promoting abstinence [18]. Bernstein et al. [21] conducted a randomized controlled trial (RCT) that provided counseling, referral to a Quitline (QL), and nicotine replacement therapy (NRT) to adult ED patients. There were significantly higher rates of abstinence and quit attempts in the intervention versus control groups. To our knowledge, only two studies have examined the efficacy of parental cessation interventions in the PED [9,11]. Although there were reductions in smoking and increased quit attempts, overall cessation rates were low. The limitations of these studies were small sample sizes, poor retention rates, and the use of behavioral counseling without the inclusion of cessation medications.

We conducted a RCT to examine the efficacy of a tobacco cessation screening, brief intervention and referral to treatment (SBIRT) plus NRT compared to an active control condition (Healthy Habits Control, HHC) delivered in the PED/UC setting. The primary outcome was parental abstinence assessed at six weeks and six months, and secondary outcomes were changes in tobacco behavior and children’s TSE. Our hypotheses were that a higher proportion of parents in the SBIRT group would be abstinent and would smoke fewer cigarettes compared to parents in the HHC group.

## 2. Materials and Methods

### 2.1. Design, Participant Recruitment and Screening

Parents presented with their child to one of two PEDs or UCs which are part of a single Midwestern tertiary care children’s hospital with over 150,000 PED/UC annual visits. The hospital Institutional Review Board approved this study. Parental participants provided written informed consent and child participants ≥age 11 provided written assent. This RCT is called Healthy Families and is registered in www.clinicaltrials.gov NCI-2531594 [23].

Clinical research coordinators (CRCs) screened a convenience sample of participants for 37 months starting in April 2016. Adults were eligible if they were ≥18 years old, spoke English, smoked combustible tobacco products daily, had a permanent address, had a working phone number, had no plans to move within the next six months, lived within a 50 mile radius of the PED/UC, and were the parent/legal guardian of a 0–17 year old patient who presented to the PED/UC with a TSE-related complaint (e.g., wheezing). Parents were excluded from enrollment into the study if: they were exclusive users of chewing tobacco or electronic cigarettes; taking smoking cessation medications; their child was an active user of cigarettes, electronic cigarettes or marijuana; or they or their child could not participate for medical or cognitive reasons.

### 2.2. Randomization and Study Conditions

Following baseline (T0) assessments, the CRC logged into a custom-built program to access the randomization scheme. SAS^®^ PROC PLAN was used to generate the stratified allocation schedule with a ratio of 1:1 using random block sizes. There were eight randomization strata: parent sex and stage of readiness to quit smoking: (1) Pre-contemplation; (2) Contemplation 1 (i.e., not planning to quit in the next 30 days; past year quit attempt); (3) Contemplation 2 (i.e., planning to quit in the next 30 days; no past year quit attempt); or (4) Preparation (i.e., planning to quit in the next 30 days; past year quit attempt) [24]. Parental sex and readiness to quit stage were entered to obtain the study condition assignment and study identification number. This study identification number was used to track the study subject over the course of the study and to ensure correct tracing of the study activities (e.g., linkage with all study samples and the associated laboratory results).

Parents were randomized to one of two conditions: (1) SBIRT plus NRT; or (2) HHC, an active control focusing on teaching parents healthy lifestyle practices for their child. Parents in the SBIRT condition received: (1) A brief, tailored MI-based coaching session on the effect TSE had on the illness that prompted their child’s PED/UC visit and how quitting may reduce their child’s TSE and subsequent illnesses; (2) Six-weeks of NRT (choice of NRT patch or NRT lozenge) for interested parents who set a quit date within 14 days and who had no contraindications [25] based on a strict study-specific checklist. If these parents used ≥80% NRT and were interested and NRT-eligible at 6 weeks after T0 (T1), they received another 6 weeks of NRT (details published elsewhere) [26]; (3) A referral to the QL during the PED/UC visit or via fax; (4) Demonstration of the smokefree.gov website and an offer to sign-up for smokefreeTXT on their phone; (5) Written materials with cessation advice and information; and (6) A weekly text message for 12 weeks with content that encouraged smoking cessation.

Parents randomized to the HHC received: (1) A brief, tailored MI-based obesity prevention coaching session that encouraged the “Let’s Go! 5-2-1-0” health practices [27,28]; (2) A water bottle; (3) Written materials on healthy diet and lifestyle choices; and (4) A weekly text message for 12-weeks with content that encouraged the 5-2-1-0 behaviors. HHC parents received no study-specific cessation treatment or TSE-related information but they could opt to get cessation information at the conclusion of the study.

The MI-based coaching sessions were given by either social workers (SW) or CRCs who had bachelor’s or master’s degrees and received MI training by an MI expert. There were nine SBIRT and 16 HHC SWs/CRCs. The median SBIRT intervention time was 11 min, including interruptions due to clinical care. SBIRT SWs/CRCs were trained as tobacco treatment specialists from certified external sites. Senior study team members trained the SW/CRCs who delivered the HHC. Both SBIRT and HHC SWs/CRCs received training and supervision by experts in behavior change interventions specific to the study conditions. All intervention sessions were audiotaped; approximately 5% of the initial audiotapes were reviewed by the principal investigator or MI expert with subsequent feedback given to SWs/CRCs.

### 2.3. Parental Assessments

At T0, parents completed assessments using REDCap (Research Electronic Data Capture [29]) on an iPad, Apple Inc, Cupertino, USA. Assessments included sociodemographics, financial strain [30] and tobacco use measures (e.g., number of cigarettes smoked by parent, number of household smokers, home smoking bans). Nicotine dependence was assessed with the heavy smoking index (HSI; range 0–6) [31,32], and motivation to quit was assessed with the contemplation ladder (range 0–10) [33].

Follow-up assessments were conducted by phone, email, or during home visits at T1 and 6-months (T2) after T0 by CRCs blinded to group assignment. Parents who self-reported that: “I haven’t smoked at all, not even a puff” received a home visit to measure exhaled carbon monoxide testing using a Bedfont MicroSmokerlyzer™, Bedfont Scientific Ltd, Kent, England, machine with ≤7 ppm as the cutoff indicating abstinence [34]. Home visits at T2 were only conducted on parents who self-reported abstinence. During study month 17, the MicroSmokerlyzer malfunctioned so salivary cotinine was subsequently obtained to confirm abstinence on participants not taking NRT; cotinine <10 ng/mL was used to indicate abstinence [34]. However, due to these inconsistencies with biochemical verification during the study, self-reported abstinence was used as the primary outcome. Secondary outcomes included: number of daily cigarettes, number of quit attempts, change in motivation to quit, participant retention, and child TSE. Parents received up to $175 for completing study procedures.

### 2.4. Child Measures, Saliva Collection and Analysis

We used two measures adapted from Wagener et al. [35], to assess parents’ perceived risk of their smoking on their child’s health: (1) perceived vulnerability (PV): perception of their child’s health risk due to parental smoking (5 items; each range 5–20) and (2) precaution effectiveness (PE): parents’ perception about the benefit on their child’s health if they quit smoking (5 items; each range 5–20). Individual items were summed to calculate the total PV and total PE score.

Child saliva samples were obtained at T0 and at the T1 home visits; saliva was obtained at T2 only if the parent reported abstinence. Saliva was tested for cotinine, a measure of recent TSE [36] using enzyme-linked immunosorbent assay (ELISA) techniques by Salimetrics LLC [23] (78.2% of T0 and all T1 and T2 samples; level of detection (LOD) = 0.15 ng/mL) or by liquid chromatography tandem mass spectrometry (LC-MS/MS) with isotope dilution (21.9% of T0 and none of the T1 and T2 samples; LOD = 0.1 ng/mL) [37]. A prior publication indicates that ELISA is a cost-effective alternative to LC-MS/MS for detecting TSE to classify children into highly exposed versus not exposed to tobacco smoke [38]. Cotinine levels less than LOD were replaced with LOD/$\sqrt{2}$. The following cotinine cut-points were used: (1) >1.0 ng/mL was used to classify children as exposed to any tobacco smoke [39]; (2) ≥3 ng/mL was equivalent to active light smoking, and (3) ≥10 ng/mL was equivalent to active smoking [34].

### 2.5. Statistical Analysis

SAS^®^ version 9.4 (SAS Institute, Cary, NC) was used for analysis. A critical value of *p* < 0.05 was considered statistically significant. Univariate and bivariate examination of variables was the first step to assess the distributional properties of the continuous outcome variables. Categorical variables are reported as *n*(%), continuous variables as mean (standard deviation (SD)) or median (interquartile range (IQR) 25th, 75th percentiles)). To control for non-normal distributions and heterogeneous error variances, logarithmic transformation was used and geometric means and associated 95% confidence intervals are reported, specifically for cotinine. Baseline variables were compared between SBIRT and HHC groups using Chi-square or Fisher’s exact test for categorical variables and t-test or Wilcoxon rank sum for continuous variables, as appropriate.

Both intent-to-treat (ITT) and complete case analyses were conducted and reported for the primary outcome of 7-day point prevalence at T1 and T2. For the ITT analysis, we assumed that those lost to follow-up were continued smokers. Trial results are reported in accordance with the CONSORT statement [40]. First, we compared the primary outcome at T1 and T2 between the SBIRT and HHC groups using Chi-square or Fisher’s exact test, as appropriate. To further assess the primary outcome adjusting for motivation to quit, logistic regression was used. For assessment of prolonged abstinence, a general linear mixed model employing general estimating equations was used due to the longitudinal nature of the analysis and also again to incorporate motivation to quit and other potential demographic covariates in the model. Care was taken with the addition of covariates in the model due to the limited number of outcome events. For secondary outcomes, a similar approach was utilized including all those with available data. Initially, a Chi-square or Fisher’s exact test for categorical variables and t-test or Wilcoxon rank sum for continuous variables was used, as appropriate. Additionally, a general linear mixed model employing general estimating equations was used to examine the longitudinal changes (T0, T1, and T2) for number of daily cigarettes, motivation to quit, number of smokers in the home, complete home smoking ban and complete home and car smoking ban and child cotinine level.

We examined potential moderators of the effects of the SBIRT condition on the primary outcomes: financial strain, NRT received at T0, and number of household smokers and we also examined potential mediators of the effects of SBIRT: PV and PE.

### 2.6. Sample Size and Power

Power analyses were based on estimates of differences in both T2 point prevalence and T2 prolonged abstinence between groups. We conservatively estimated a point prevalence cessation rate of 6.3% and prolonged abstinence rate of 2.1% in the HHC group, based on prior research [41]. We based our estimate of T2 point prevalence of the SBIRT group on the odds ratio derived from meta-analyses assessing the effectiveness of NRT vs. a control, ranging from 1.4 to 4.65 to give a cessation rate of 10–12% in the SBIRT group [42], since we expected high compliance with a 12% estimated cessation rate in the SBIRT group. Similarly, for prolonged abstinence, we assumed a rate of 7% in the SBIRT group. With 300 subjects in each group, we would have 80% power to detect an increase of 6.4% in cessation rate. For T2 abstinence, we would be able to detect an increase of 4.5% with 300 subjects per group.

## 3. Results

### 3.1. Participant Characteristics

**Figure 1:**
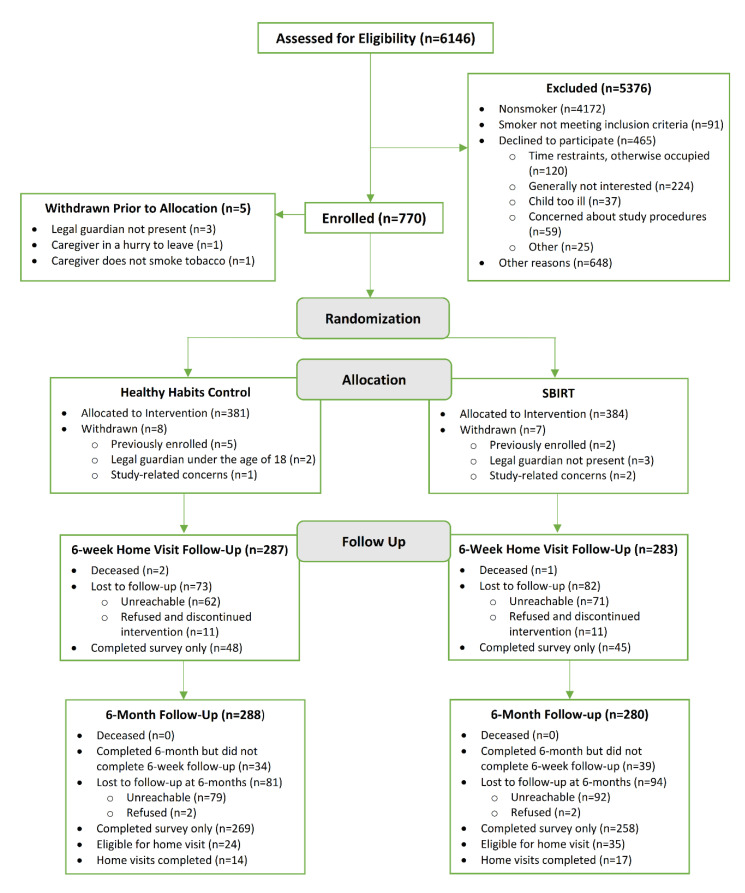
Flow of participants through the study.

From April 2016 to May 2019, 750 parents were enrolled. See Figure 1 and Table 1, respectively, for the study flow diagram and baseline characteristics of participants. The mean (SD) age was 31.8 (7.7) years; the majority were female (86.8%), Black (52.7%), had public or no insurance (87.3%; 3.6% had no insurance), and had an income level of ≤$15,000 (64.6%). The median (IQR) number of daily cigarettes smoked were 10 [5,15]. At T0, there were no significant differences between the SBIRT and HHC groups. Follow-up assessments were completed by email or phone on 76% of participants at T2. Those who completed follow-up assessments at T2 and thus were successfully retained were more likely to be female (88.2% vs. 82.3%, *p* = 0.04), Black (55.3% vs. 44.8%, *p* = 0.01), and have more than a high school education (64.1% vs. 54.3%, *p* = 0.03).

### 3.2. Child T0 Cotinine Levels

Children were highly exposed to tobacco smoke; only 67 (8.9%) were considered not/minimally exposed based on cotinine <1.0 ng/mL [39]. Cotinine levels ranged from 0.07 to 363.78 ng/mL; geometric mean (95% CI) was 5.57 (5.05, 6.16) ng/mL, median (IQR) was 6.10 (2.61, 12.05) ng/mL. A total of 70.8% of children had cotinine ≥3.0 ng/mL, and 30.8% had cotinine ≥10 ng/mL. There were no differences in cotinine levels between conditions.

### 3.3. Primary and Secondary Outcomes at T1 and T2

Overall quit rates using complete case analysis were 4.2% in both groups at T1 and 12.9% and 8.3% in the SBIRT and HHC groups, respectively. Using ITT, quit rates were 9.5% and 6.4% in the SBIRT and HHC groups, respectively, at T2. The GLMM analysis including both T1 and T2 as ITT yielded similar non-significant results with an odds ratio of 1.34 (95% confidence interval (CI): 0.84, 2.15, *p* = 0.22). Invoking GEE, the odds ratio was 1.40 (95% CI 0.87, 2.25; *p* = 0.16). Since the primary outcomes were non-significant, further ITT analyses were not conducted.

Using complete case analyses, there was a statistically significant difference in the SBIRT compared to the HHC group on the following secondary outcomes: median [IQR] reduction of daily cigarettes smoked by SBIRT compared to HHC participants at T1 compared to T0 (−2 [−5, 0] vs 0 [−4, 0], *p* = 0.0008) and at T2 compared to T0 (−4 [−9, −1] vs −2 [−5, 0], *p* = 0.0006), and increased mean (SD) number of quit attempts at T2 compared to T0 (1.25 (6.5) vs. 0.02 (4.71), *p* = 0.02). See Table 2.

Findings for secondary outcomes using a GLMM analysis were as follows: for number of cigarettes smoked there was a significant interaction term for group by time: *p* = 0.03, with a specific difference at T2 between SBIRT and HHC; for motivation to quit there was a significant interaction group by time effect (*p* = 0.02), but no specific differences were found between SBIRT and HHC; there was no significant group by time interaction for number of smokers in the home (*p* = 0.35); there was no significant group by time interaction for complete home smoking ban or complete home and car smoking ban (*p* = 0.72 and *p* = 0.13, respectively). The individual differences are displayed in Table 2.

There were no statistically significant differences in the number of home and car smoking bans at T1 or T2. There were no statistically significant differences in cotinine levels in children of SBIRT or HHC parents from T0 to T1 or T2. See Table 2.

Table 3 shows the self-reported quit rates in pre-specified potential moderators and mediators. The following statistically significant differences were found: higher quit rates at T1 in SBIRT parents who received NRT (83.3% vs. 50.9%, *p* = 0.04) but not at T2; lower quit rates at T1 in parents who had >1 household smoker (37.5% vs. 71.1%, *p* = 0.001); and higher quit rates in parents with higher PE at T2 (73.3% vs. 55.2%, *p* = 0.007).

### 3.4. Cessation Resources Given to SBIRT Participants

All SBIRT participants received MI-based cessation coaching and 366 (97%) received at least one cessation resource. Of these, 345 (94%) received QL information, and 50 (14%) accepted fax QL referral. A total of 354 (97%) parents were referred to smokefreeTXT and 23 (6%) signed up for the program during the PED/UC visit. A total of 361 (96%) were given smokefree.gov information and 170 (47%) visited the website during their visit. A total of 252 (66.8%) were interested in receiving NRT and willing to set a quit date within 14 days; 53 (21%) interested parents had >1 NRT contraindication. Thus, 199 (52.8%) participants received 6 weeks of NRT in the PED/UC [26]. Notably, 329 (88%) HHC participants requested smoking cessation information at the end of the study.

## 4. Discussion

This RCT was designed to leverage the pediatric emergency visit to provide a public health intervention that would benefit both parents and children. We tested the efficacy of an SBIRT with NRT to achieve tobacco abstinence in parental smokers and TSE reduction in their children compared to an attention control condition. Similar to an adult ED-based study [20], our results suggest that parental smokers in the PED/UC setting are interested in receiving help with quitting and that they took important steps towards abstinence.

We observed decreases in the median number of daily cigarettes smoked and increased quit attempts in SBIRT parents. Overall, these findings indicate that the SBIRT model resulted in positive changes in parental smokers’ tobacco use and that the SBIRT model could decrease children’s TSE. Any and all quit attempts and decreases in the number of cigarettes smoked benefits both parents and children and represents an opportunity that may eventually result in abstinence [43].

There are several possible explanations for the lack of significant differences in abstinence between conditions. First, it was challenging to consistently provide each of the intervention components in a uniform fashion in terms of time and “dose” of each component due to the fast pace of the PED/UC visit and the vast range of clinical acuity in the children. The median SBIRT length was only 11 min. While we sought to leverage parental concern about their child’s illness during the PED/UC visit as a “teachable moment” to mobilize parents to quit smoking, we found no differences in cessation outcomes based on parental reports of PV or PE at T0. This may have been due to the brief assessments used to assess parental PV and PE which may not have revealed parents’ actual feelings about how smoking affects their child. These questions along with qualitative interviews should be conducted in future trials to reveal the types of anxiety and specific concerns parents have in this setting which may further motivate them (or deter them) to take steps to quit after the PED/UC visit. Another issue was high SW/CRC turnover rates and the varied types of prior education and/or training that the SWs/CRCs had which may have also contributed to lower rates of cessation. Further, few participants used additional cessation resources, and only 52.8% of SBIRT parents received NRT due strict eligibility criteria. Future pediatric-based trials should consider giving NRT to adult smokers with hypertension, for example, since this is not a contraindication for NRT in adult ED-based trials [21,44]. However, it is encouraging that SBIRT parents who did receive NRT had higher rates of quitting. Further research is needed on how to provide more consistent intervention delivery including NRT in the PED/UC setting.

Second, there were higher-than-expected quit rates in HHC parents, which could be due to higher-than-expected readiness to quit in this group. Indeed, this is reflected by the finding that 85% of parents were in the contemplation or preparation stage of cessation at T0 [24] and 88% of HHC parents requested cessation information at T2. Although the HHC condition did not contain information or guidance on tobacco use, the health messages inherent in the condition may have indirectly activated parents to reduce smoking.

Third, study results may have been biased in that the T0 assessment had 62 questions of which 47 (76%) were about tobacco use/TSE. This could have resulted in “assessment reactivity [45]” as the assessment may have provided both the SBIRT and HHC participants a “dose” of the intervention, time to think about their tobacco use and the effects of TSE on their child, and the impetus to quit smoking after enrollment. Similar findings and concerns have been reported in other ED-based trials [20,21,46,47]. Further, the follow-up assessments about tobacco use and TSE may have provided additional prompts for parents to change their smoking behavior.

Finally, other ED-based studies that have achieved statistically significant abstinence rates employed strategies that we did not use, including booster telephone calls, administration of the first NRT dose in the ED, and dual modalities of NRT with both longer-acting NRT patches and shorter-acting NRT gum [44,48,49]. Future PED/UC studies should explore adding these components. Similarly, the lack of observed differences in smoking bans or child cotinine levels may be due to our brief intervention with no booster coaching. A recent review of prior child TSE reduction interventions reported that 33% of 78 studies had successful reductions in child TSE [17]. The successful trials used more intensive interventions than we employed. Thus, future work should include follow-up counseling and messaging to ensure that participants have the greatest chance to quit and reduce their children’s TSE.

This study had several strengths. The sample size was large, an attention control condition was compared to the SBIRT condition, NRT was administered to parents who were not patients, six-month follow-up assessments were conducted, biochemical assessments were obtained, and data were gathered from multiple sources. The conduct of this study also speaks to the feasibility of incorporating tobacco interventions in this busy setting. However, aspects of this study limit the generalizability of findings. It was conducted at a single, midwestern tertiary care PED/UC. Although our predominantly low-income study population had diverse racial/ethnic backgrounds, the majority of parents were female which is common in smoking cessation interventions in the pediatric setting [17,50,51]. It is unknown if similar results would have been obtained in a different setting or with a different study population. Further, the intervention was conducted by trained, non-clinical research staff, and we experienced high turnover. Interventions had limited face-to-face time and it is possible that improved results may have been observed if clinical staff had delivered the SBIRT during the course of the PED/UC visit using digital or other tools. The use of clinical decision support tools may facilitate the delivery of cessation intervention components by clinical staff based on studies conducted in the PED, UC, and primary care settings [52,53,54]. Additionally, many state QLs have electronic referral options [55] which eases the burden on clinicians and may improve cessation outcomes after the PED/UC visit. More research is needed in this area.

## 5. Conclusions

A PED/UC intervention decreased the number of cigarettes smoked, increased quit attempts, and resulted in higher rates of quitting in parents who received NRT. Although significant differences in quit rates across conditions were not found, the SBIRT condition was successful in facilitating quit attempts, changes in smoking behavior, and short-term abstinence, all of which are essential in helping smokers achieve long-term cessation [56,57]. The potential benefit of such cessation interventions, defined as reach (number exposed to the intervention) multiplied by efficacy (percentage who quit), could have a substantial public health impact [58]. For example, if 30% of the parents who bring their children to EDs annually are smokers, then approximately nine million parental smokers could be treated with brief interventions [1]. If 10% of these smokers quit, this would equal approximately 900,000 new ex-smokers annually. Numbers would be higher if UC visits were also utilized. Our study represents an important foundation on which to launch future cessation and TSE reduction research in the PED/UC setting.

## Figures and Tables

**Table 1:** Characteristics of the study population overall and by study group.

Variable	Overall	SBIRT ^1^	HHC ^2^	*p*-Value
	(*N* = 750)	(*N* = 377)	(*N* = 373)	SBIRT vs. HHC
	*n* (%)	*n* (%)	*n* (%)	
Parent Age, Mean (SD)	31.76 (7.65)	31.97 (8.23)	31.55 (7.03)	0.46 *
Parent Sex-Female	651 (86.80)	330 (87.53)	321 (86.06)	0.55
Parent Race/Ethnicity				0.99 **
White, non-Hispanic	285 (39.39)	149 (39.52)	146 (39.25)	
Black, non-Hispanic	395 (52.74)	198 (52.52)	197 (52.96)	
Other, non-Hispanic	42 (5.61)	22 (5.84)	20 (5.38)	
Hispanic	17 (2.27)	8 (2.12)	9 (2.42)	
Insurance				0.75
Public or None	655 (87.33)	328 (87.00)	327 (87.67)	
Commercial	80 (10.67)	40 (10.61)	40 (10.72)	
Other	15 (2.00)	9 (2.39)	6 (1.61)	
Income				0.06
≤$15,000	483 (64.57)	231 (61.27)	252 (67.92)	
>$15,000	265 (35.43)	146 (38.73)	119 (32.08)	
Financial Strain				0.17 *
Mean (SD)	2.41 (1.10)	2.46 (1.10)	2.35 (1.10)	
Median (IQR)	2.33 (1.67, 3.00)	2.33 (1.67, 3.33)	2.00 (1.33, 3.00)	
Employed (yes)	403 (54.24)	208 (56.06)	195 (52.42)	0.32
Highest Education Level				0.43
Less than HS/HS	427 (56.93)	220 (58.36)	207 (55.50)	
≥Some college	323 (43.07)	157 (41.64)	166 (44.50)	
Child age, Mean (SD)	4.91 (4.71)	5.07 (4.88)	4.74 (5.54)	0.36 *
Number of Daily Cigarettes Smoked by the Parent				0.39
Mean (SD)	10.23 (7.23)	10.53 (7.47)	9.93 (6.97)	
Median (IQR)	10.00 (5, 15)	10.00 (5, 15)	10.00 (5, 15)	
Current electronic cigarette user	39 (5.20)	18 (4.83)	21 (5.57)	0.65
Stage of Change				0.94
Pre-Contemplation	111 (14.80)	56 (14.85)	55 (14.75)	
Contemplation	402 (53.60)	204 (54.11)	198 (53.08)	
Preparation	237 (31.60)	117 (31.03)	120 (32.17)	
Past Year Quit Attempts	539 (71.87)	262 (69.50)	277 (74.26)	0.15
Number of Household SmokersMean (SD)	2.29 (1.45)	2.53 (1.48)	2.44 (1.42)	0.41 *
Home Smoking Ban	212/485 (43.71)	107/243 (44.03)	105/242 (43.39)	0.89
Home and Car Ban	93/485 (19.18)	44/243 (18.11)	49/242 (20.25)	0.55
Cotinine ng/mLMedian (IQR)	6.10 (2.61, 12.05)	6.74 (2.70, 12.58)	5.60 (2.40, 11.65)	0.40 *
Parent Believes Smoking Affects Child’s Health (PV ^3^ total), Mean (SD)	11.95 (4.22)	11.93 (4.16)	11.97 (4.28)	0.90 *
Parent Believes Quitting Would Benefit Child (PE ^4^ total), Mean (SD)	13.35 (4.71)	13.21 (4.70)	13.49 (4.72)	0.43 *

Notes: ^1^ SBIRT= Screening, Brief Intervention, Referral to Treatment; ^2^ HHC = Healthy Habit Control; ^3^ PV = Perceived Vulnerability; ^4^ PE = Precaution Effectiveness; * Tested using Wilcoxon rank sum; ** Tested using Fisher’s exact test; Missing data: Smoking ban (*n* = 265); T0 cotinine was assessed on *n* = 346 samples in SBIRT and *n* = 345 samples in HHC groups.

**Table 2:** Primary and secondary outcomes at T1 ^1^ and T2 ^2^.

Variable	SBIRT^3^	HHC^4^	*p*-Value	SBIRT	HHC	*p*-Value
	T1	T1		T2	T2	
	*n (%)*	*n (%)*		*n (%)*	*n (%)*	
*N*	283	288	--	280	289	--
Abstinence, Biochemically Verified *N*/ Total *N* of Participants Who Self-Reported Quitting (%)	9/10 (90)	5/8 (62.5)	0.27 ^+^	11/17 (64.7)	10/14 (71.4)	1.00 ^+^
Abstinence, Self-report	12 (4.24)	12 (4.17)	0.96	36 (12.9)	24 (8.30)	0.08
Number of Daily CigarettesMedian (IQR)	6 (3, 10)	7 (4, 11.5)	0.11	5 (2, 10)	5 (2, 10)	**0.049**
Change in Number of Daily Cigarettes from Baseline Median (IQR)	−2 [−5, 0]	0 [−4, 0]	**0.0008**	−4 [−9, −1]	−2 [−5, 0]	**0.0006**
Change in Number of Daily Cigarettes from T1 Median (IQR)	--	--	--	0 [−4, 1]	0 [−2, 0]	0.84
Motivation to Quit			**0.03**			
Mean (SD)	6.98 (2.47)	6.46 (2.78)		7.03 (2.58)	6.77 (2.80)	0.41
Median (IQR)	8 (5, 9)	6 (5, 9)		8 (5, 9)	7 (5, 9)	
Quit Attempts Since Baseline						
Mean (SD)	2.69 (4.98)	2.22 (2.53)	0.99	3.82 (6.36)	2.34 (3.24)	**0.003**
Median (IQR)	2 (0,3)	2 (0, 3)		2 (1, 4)	2 (0, 3)	
Change in Number of Quit Attempts from Baseline			0.11			**0.02**
Mean (SD)	0.44 (5.54)	−0.25 (4.85)		1.25 (6.5)	0.02 (4.71)	
Median (IQR)	0 (−1,1)	0 (−1, 1)		0 (−2, 1)	0 (−1, 1)	
Number of Household Smokers						
Mean (SD)	1.49 (1.28)	1.45 (0.76)	0.9	1.22 (0.80)	1.21 (0.64)	
Median (IQR)	1 (1, 2)	1 (1, 2)		1 (1, 1)	1 (1, 2)	0.59
Home Smoking Ban	82 (43.85)	82 (44.09)	0.96	111 (51.63)	120 (52.63)	0.83
Home and Car Smoking Ban	47 (25.13)	38 (20.32)	0.27	69 (32.09)	84 (36.84)	0.29
Cotinine Change from T0 ng/mL *			0.05			0.66
Median (IQR)	1.81 (−2.94, 3.24)	1.81 (−2.02, 5.52)		2.44 (−1.61, 6.92)	1.81 (−2.18, 5.52)	

Notes: ^1^ T1 = 6-weeks; ^2^ T2 = 6-months; ^3^ SBIRT= Screening, Brief Intervention, Referral to Treatment; ^4^ HHC = Healthy Habit Control; ^+^ Fisher’s exact test; * T1 cotinine was assessed on *n* = 167 samples in SBIRT and *n* = 147 samples in HHC groups; T2 cotinine was assessed in *n* = 38 samples in SBIRT and *n* = 26 samples in HHC groups; Boldface print indicates *p* < 0.05.

**Table 3:** Self-reported abstinence at T1 ^1^ and T2 ^2^ for specific subgroups.

Variable	Yes, at T1	No, at T1	*p*-Value	Yes, at T2	No, at T2	*p*-Value
	*n (%)*	*n (%)*		*n (%)*	*n (%)*	
*N*	24	547		60	509	
Financial Strain >2 at Baseline	11 (45.83)	201 (53.20)	0.53 *	27 (45.00)	267 (52.46)	0.27
NRT ^3^ Given at Baseline ^†^	10/12 (83.33)	138/271 (50.92)	0.04 *	20/36 (55.56)	124/244 (50.82)	0.60
Number of Household Cigarette Smokers >1	9 (37.50)	389 (71.12)	0.001 *	43 (71.67)	353 (69.35)	0.71
Parent Believes Smoking Affects Child’s Health (PV ^4^ ≥12) at Baseline	16 (66.67)	288 (52.65)	0.21 *	31 (51.67)	263 (51.67)	1.00
Parent Believes Quitting Would Benefit Child (PE ^5^ ≥14) at Baseline	13 (54.17)	270 (49.36)	0.68 *	32 (53.33)	254 (49.90)	0.62
Parent Believes Smoking Affects Child’s Health (PV ≥ 12) at T1	16 (66.67)	292/544 (53.68)	0.30 *	23/47 (48.94)	247/446 (55.38)	0.40
Parent Believes Quitting Would Benefit Child (PE ≥ 14) at T1	15 (62.50)	277/544 (50.92)	0.30 *	26/47 (55.32)	229/446 (51.35)	0.60
Parent Believes Smoking Affects Child’s Health (PV ≥ 12) at T2	11/20 (55.00)	253/476 (53.15)	1.00 *	34 (56.67)	265 (52.06)	0.50
Parent Believes Quitting Would Benefit Child (PE ≥ 14) at T2	14/20 (70.00)	261/476 (54.83)	0.25 *	44 (73.33)	281 (55.21)	0.007

Notes: ^1^ T1 = 6-weeks; ^2^ T2 = 6-months; ^3^ NRT = Nicotine Replacement Therapy; ^4^ PV = Perceived Vulnerability; ^5^ PE = Precaution Effectiveness; ^†^ SBIRT only; * Fisher’s exact test; Boldface print indicates *p* < 0.05.

## References

[1] McDermott K., Stocks C., Freeman W. (2018). Statistical Brief #242. Healthcare Cost and Utilization Project (HCUP).

[2] Committee on Pediatric Emergency Medicine (2014). Pediatric care recommendations for freestanding urgent care facilities. Pediatrics.

[3] Bornais J.A.K., Crawley J., El-Masri M.M. (2020). One Stop: Examining the Reasons Patients Use the Emergency Department for Nonurgent Care and the Barriers They Face. J. Emerg. Nurs..

[4] Farion K.J., Wright M., Zemek R., Neto G., Karwowska A., Tse S., Reid S., Jabbour M., Poirier S., Moreau K.A. (2015). Understanding Low-Acuity Visits to the Pediatric Emergency Department. PLoS ONE.

[5] Long C.M., Mehrhoff C., Abdel-Latief E., Rech M., Laubham M. (2018). Factors Influencing Pediatric Emergency Department Visits for Low-Acuity Conditions. Pediatr. Emerg. Care.

[6] Schlichting L.E., Rogers M.L., Gjelsvik A., Linakis J.G., Vivier P.M. (2017). Pediatric Emergency Department Utilization and Reliance by Insurance Coverage in the United States. Acad. Emerg. Med..

[7] Orlansky A., Smolij C., Moughan B., Aronoff S.C. (2016). Factors that Affect Nonurgent Emergency Department Visits in a Publicly Insured Pediatric Population: An Observational Study. J. Healthc. Qual..

[8] Burns R.R., Alpern E.R., Rodean J., Canares T., Lee B.R., Hall M., Montalbano A. (2020). Factors Associated With Urgent Care Reliance and Outpatient Health Care Use Among Children Enrolled in Medicaid. JAMA Netw. Open.

[9] Mahabee-Gittens E.M., Khoury J.C., Ho M., Stone L., Gordon J.S. (2015). A smoking cessation intervention for low-income smokers in the ED. Am. J. Emerg. Med..

[10] Mahabee-Gittens E.M., Stone L., Gordon J.S. (2013). Pediatric emergency department is a promising venue for adult tobacco cessation interventions. Nicotine Tob. Res..

[11] Mahabee-Gittens E.M., Gordon J.S., Krugh M.E., Henry B., Leonard A.C. (2008). A smoking cessation intervention plus proactive quitline referral in the pediatric emergency department: A pilot study. Nicotine Tob. Res..

[12] Hall N., Hipple B., Friebely J., Ossip D.J., Winickoff J.P. (2009). Addressing Family Smoking in Child Health Care Settings. J. Clin. Outcomes Manag..

[13] Mahabee-Gittens E.M., Gordon J. (2008). Acceptability of tobacco cessation interventions in the pediatric emergency department. Pediatr. Emerg. Care.

[14] Mahabee-Gittens E.M., Merianos A.L., Stone L., Tabangin M.E., Khoury J.C., Gordon J.S. (2019). Tobacco Use Behaviors and Perceptions of Parental Smokers in the Emergency Department Setting. Tob. Use Insights.

[15] Ralston S., Roohi M. (2008). A randomized, controlled trial of smoking cessation counseling provided during child hospitalization for respiratory illness. Pediatr. Pulmonol..

[16] Kells M., Rogers J., Oppenheimer S.C., Blaine K., McCabe M., McGrath E., Woodring B., Geller A.C. (2013). The teachable moment captured: A framework for nurse-led smoking cessation interventions for parents of hospitalized children. Public Health Nurs..

[17] Behbod B., Sharma M., Baxi R., Roseby R., Webster P. (2018). Family and carer smoking control programmes for reducing children’s exposure to environmental tobacco smoke. Cochrane Database Syst. Rev..

[18] Lemhoefer C., Rabe G.L., Wellmann J., Bernstein S.L., Cheung K.W., McCarthy W.J., Lauridsen S.V., Spies C., Neuner B. (2017). Emergency Department-Initiated Tobacco Control: Update of a Systematic Review and Meta-Analysis of Randomized Controlled Trials. Prev. Chronic. Dis..

[19] Rabe G.L., Wellmann J., Bagos P., Busch M.A., Hense H.W., Spies C., Weiss-Gerlach E., McCarthy W., Gareca Arizaga M.J., Neuner B. (2013). Efficacy of emergency department-initiated tobacco control—Systematic review and meta-analysis of randomized controlled trials. Nicotine Tob. Res..

[20] Bernstein S.L., Bijur P., Cooperman N., Jearld S., Arnsten J.H., Moadel A., Gallagher E.J. (2011). A randomized trial of a multicomponent cessation strategy for emergency department smokers. Acad. Emerg. Med..

[21] Bernstein S.L., D’Onofrio G., Rosner J., O’Malley S., Makuch R., Busch S., Pantalon M.V., Toll B. (2015). Successful Tobacco Dependence Treatment in Low-Income Emergency Department Patients: A Randomized Trial. Ann. Emerg. Med..

[22] Bernstein S.L., Rosner J., Toll B. (2016). A Multicomponent Intervention Including Texting to Promote Tobacco Abstinence in Emergency Department Smokers: A Pilot Study. Acad. Emerg. Med..

[23] Mahabee-Gittens E.M., Ammerman R.T., Khoury J., Stone L., Meyers G.T., Witry J.K., Merianos A.L., Mancuso T.F., Stackpole K., Bennett B.L. (2017). Healthy families: Study protocol for a randomized controlled trial of a screening, brief intervention, and referral to treatment intervention for caregivers to reduce secondhand smoke exposure among pediatric emergency patients. BMC Public Health.

[24] Prochaska J.O., DiClemente C.C., Norcross J.C. (1992). In search of how people change. Applications to addictive behaviors. Am. Psychol..

[25] Fiore M., Jaen C., Baker T., Bailey W., Benowitz N.L., Curry S.J., Dorfman S.F., Froelicher E.S., Goldstein M.G., Healton C.G. (2008). Treating Tobacco Use and Dependence: 2008 Update.

[26] Mahabee-Gittens E.M., Merianos A.L., Tabangin M.E., Stone L., Gordon J.S., Khoury J.C. (2020). Provision of free nicotine replacement therapy to parental smokers in the pediatric emergency setting. Tob. Prev. Cessat..

[27] Foltz J.L., Cook S.R., Szilagyi P.G., Auinger P., Stewart P.A., Bucher S., Dipl D., Baldwin C.D. (2011). US Adolescent Nutrition, Exercise, and Screen Time Baseline Levels Prior to National Recommendations. Clin. Pediatr..

[28] Rogers V.W., Hart P.H., Motyka E., Rines E.N., Vine J., Deatrick D.A. (2013). Impact of Let’s Go! 5-2-1-0: A Community-Based, Multisetting Childhood Obesity Prevention Program. J. Pediatr. Psychol..

[29] Harris P.A., Taylor R., Thielke R., Payne J., Gonzalez N., Conde J.G. (2009). Research electronic data capture (REDCap)—A metadata-driven methodology and workflow process for providing translational research informatics support. J. Biomed. Inform..

[30] Vinokur A.D., Price R.H., Caplan R.D. (1996). Hard times and hurtful partners: How financial strain affects depression and relationship satisfaction of unemployed persons and their spouses. J. Personal. Soc. Psychol..

[31] Pérez-Ríos M., I Santiago-Pérez M., Alonso B., Malvar A., Hervada X., De Leon J. (2009). Fagerstrom test for nicotine dependence vs heavy smoking index in a general population survey. BMC Public Health.

[32] Chabrol H., Niezborala M., Chastan E., De Leon J. (2005). Comparison of the Heavy Smoking Index and of the Fagerstrom Test for Nicotine Dependence in a sample of 749 cigarette smokers. Addict. Behav..

[33] Biener L., Abrams D.B. (1991). The Contemplation Ladder: Validation of a measure of readiness to consider smoking cessation. Health Psychol..

[34] Benowitz N.L., Bernert J.T., Foulds J., Hecht S.S., Jacob P., Jarvis M.J., Joseph A., Oncken C., Piper M.E. (2019). Biochemical Verification of Tobacco Use and Abstinence: 2019 Update. Nicotine Tob. Res..

[35] Wagener T.L., Gregor K.L., Busch A.M., McQuaid E.L., Borrelli B. (2010). Risk perception in smokers with children with asthma. J. Consult. Clin. Psychol..

[36] Benowitz N.L., Hukkanen J., Jacob P. (2009). Nicotine Chemistry, Metabolism, Kinetics and Biomarkers. Handb. Exp. Pharmacol..

[37] Murphy S.E., Wickham K.M., Lindgren B.R., Spector L.G., Joseph A.M. (2013). Cotinine and trans 3′-hydroxycotinine in dried blood spots as biomarkers of tobacco exposure and nicotine metabolism. J. Expo. Sci. Environ. Epidemiol..

[38] Mahabee-Gittens E.M., Mazzella M.J., Doucette J.T., Merianos A.L., Stone L., Wullenweber C.A., Busgang S.A., Matt G.E. (2020). Comparison of Liquid Chromatography Mass Spectrometry and Enzyme-Linked Immunosorbent Assay Methods to Measure Salivary Cotinine Levels in Ill Children. Int. J. Environ. Res. Public Health.

[39] Butz A., Tsoukleris M., Bollinger M.E., Jassal M., Bellin M.H., Kub J., Mudd S., Ogborn C.J., Lewis-Land C., Thompson R.E. (2019). Association between second hand smoke (SHS) exposure and caregiver stress in children with poorly controlled asthma. J. Asthma.

[40] Schulz K.F., Altman D.G., Moher D. (2010). Consort Group, CONSORT 2010 Statement: Updated guidelines for reporting parallel group randomised trials. BMC Med..

[41] Gordon J.S., Andrews J.A., Albert D.A., Crews K.M., Payne T.J., Severson H.H. (2010). Tobacco Cessation via Public Dental Clinics: Results of a Randomized Trial. Am. J. Public Health.

[42] Stead L.F., Perera R., Bullen C., Mant D., Hartmann-Boyce J., Cahill K., Lancaster T. (2012). Nicotine replacement therapy for smoking cessation. Cochrane Database Syst. Rev..

[43] Bernstein S.L., Toll B.A. (2019). Ask about smoking, not quitting: A chronic disease approach to assessing and treating tobacco use. Addict. Sci. Clin. Pract..

[44] Bernstein S., Dziura J., Weiss J., Harper Brooks A., Miller T., Vickerman K., Grau L., Pantalon M., Abroms L., Collins L. (2020). Successful Treatment of Tobacco Dependence Treatment in the Emergency Department Using the Multiphase Optimization Strategy. Annual Meeting of the Society for Research on Nicotine and Tobacco.

[45] Bernstein J., Bernstein E., Heeren T.C. (2010). Mechanisms of change in control group drinking in clinical trials of brief alcohol intervention: Implications for bias toward the null. Drug Alcohol Rev..

[46] Bernstein E., Bernstein J. (2008). Effectiveness of alcohol screening and brief motivational intervention in the emergency department setting. Ann. Emerg. Med..

[47] D’Onofrio G., Pantalon M.V., DeGutis L.C., Fiellin D.A., Busch S.H., Chawarski M.C., Owens P.H., O’Connor P.G. (2008). Brief Intervention for Hazardous and Harmful Drinkers in the Emergency Department. Ann. Emerg. Med..

[48] Bernstein S.L., Bijur P., Cooperman N.A., Jearld S., Arnsten J.H., Moadel A., Gallagher E.J. (2013). Efficacy of an emergency department-based multicomponent intervention for smokers with substance use disorders. J. Subst. Abus. Treat..

[49] Bock B.C., Becker B.M., Niaura R., Partridge R., Fava J.L., Trask P. (2008). Smoking cessation among patients in an emergency chest pain observation unit: Outcomes of the Chest Pain Smoking Study (CPSS). Nicotine Tob. Res..

[50] Nabi-Burza E., Drehmer J.E., Walters B.H., Rigotti N.A., Ossip D.J., Levy D.E., Klein J.D., Regan S., Gorzkowski J.A., Winickoff J.P. (2019). Treating Parents for Tobacco Use in the Pediatric Setting. JAMA Pediatr..

[51] Collins B.N., Lepore S.J., Winickoff J.P., Sosnowski D.W. (2019). Parents’ self-efficacy for tobacco exposure protection and smoking abstinence mediate treatment effects on child cotinine at 12-month follow-up: Mediation results from the Kids Safe and Smokefree trial. Nicotine Tob. Res..

[52] Mahabee-Gittens E.M., Merianos A.L., Dexheimer J.W., Meyers G.T., Stone L., Tabangin M., Khoury J.C., Gordon J.S. (2018). Utilization of a Clinical Decision Support Tool to Reduce Child Tobacco Smoke Exposure in the Urgent Care Setting. Pediatr. Emerg. Care.

[53] Mahabee-Gittens E.M., Dexheimer J.W., Tabangin M., Khoury J.C., Merianos A.L., Stone L., Meyers G.T., Gordon J.S. (2018). An Electronic Health Record−Based Strategy to Address Child Tobacco Smoke Exposure. Am. J. Prev. Med..

[54] Jenssen B.P., Muthu N., Kelly M.K., Baca H., Shults J., Grundmeier R.W., Fiks A.G. (2019). Parent eReferral to Tobacco Quitline: A Pragmatic Randomized Trial in Pediatric Primary Care. Am. J. Prev. Med..

[55] Keller P.A., Lien R.K., Beebe L.A., Parker J., Klein P., Lachter R.B., Gillaspy S. (2020). Replicating state Quitline innovations to increase reach: Findings from three states. BMC Public Health.

[56] Kruse G.R., Park E.R., Chang Y., Haberer J.E., Abroms L.C., Shahid N.N., Howard S., Haas J.S., Rigotti N.A. (2020). Proactively Offered Text Messages and Mailed Nicotine Replacement Therapy for Smokers in Primary Care Practices: A Pilot Randomized Trial. Nicotine Tob. Res..

[57] Chaiton M., Diemert L., Cohen J.E., Bondy S.J., Selby P., Philipneri A., Schwartz R. (2016). Estimating the number of quit attempts it takes to quit smoking successfully in a longitudinal cohort of smokers. BMJ Open.

[58] Bernstein S.L., Boudreaux E.D., Cydulka R.K., Rhodes K.V., Lettman N.A., Almeida S.L., McCullough L.B., Mizouni S., Kellermann A.L. (2006). American College of Emergency Physicians Task Force on Smoking, C. Tobacco control interventions in the emergency department: A joint statement of emergency medicine organizations. Ann. Emerg. Med..

